# Idiopathic Polypoidal Choroidal Vasculopathy in a Young Man: Case Report and Literature Review

**DOI:** 10.4103/0974-9233.52000

**Published:** 2008

**Authors:** Saba Al-Rashaed

**Affiliations:** From Vitreoretinal Division, King Khaled Eye Specialist Hospital, Riyadh, Saudi Arabia

**Keywords:** idiopathic polypoidal choroidal vasculopathy, severe visual loss, photodynamic therapy

## Abstract

**Purpose:**

To report a case of idiopathic polypoidal choroidal vasculopathy and its angiographic characteristics in a young man.

**Method:**

Clinical data including visual acuity, color fundus photography, fluorescein angiograph and indocyanine green findings and management of this case are presented.

**Results:**

A young healthy male was presented with sudden loss of vision in the left eye for 3 weeks duration and a visual of 1/200. Fundus examination of the eye showed vitreous hemorrhage with massive sub retinal yellowish old blood in the posterior pole with epiretinal fresh blood on the fovea. Intra-venous Fluorescein angiography and Indocyanine green tests showed features of Idiopathic Polypoidal Choroidal Vasculopathy. The patient received photodynamic therapy twice without significant visual improvement.

**Conclusion:**

Idiopathic Polypoidal Choroidal Vasculopathy can occur in a young age group and can lead to severe visual impairment despite treatment with PDT.

Idiopathic Polypoidal Choroidal Vasculopathy (IPCV), as a cause of recurrent hemorrhagic and oxidative retinal pigment epithelium (RPE) and neurosensory retina detachment, was first described by Yanuzzi.^1^ In the past, a variety of terms such as ‘posterior uveal bleeding syndrome’^2^,^3^ and ‘multiple recurrent retinal pigment epithelium detachment’ in black hypertensive women have been used to designate this disorder.^4^,^5^ The primary abnormality typically involves the choroidal circulation and the characteristic lesions is present in the inner choroidal vascular network of vessels ending in an aneurismal bulge or outward projection visible clinically as a reddish orange spheroidal polyp-like structure. It is associated with multiple, recurrent, serosenguineous detachment of RPE and neurosensory retina – secondary to leakage and bleeding from the peculiar choroidal vascular lesion. Vitreous hemorrhage, relatively minimal scarring and absence of drusen, retinal vascular disease and signs of intraocular inflammation are also reported in the literature.^1^,^6^,^7^

The diagnosis of IPCV can best be established using indocyanine green test (ICG) as it permits visualization of the choroids' vasculature and can show the typical aneurismal and spheroidal vascular dilation of the choroidal vessels.^7^

The disease onset was typically seen among the elderly population. The reported average age of the onset of IPCV was 60 years which is significantly younger in age than related macular degeneration.^8^ Yannuzi et al reported a case in which clinical manifestation was seen in a 20-year-old patient, and to the best of our knowledge this will be the second reported case in a young patient.

## Case Report

**Figure 1:**
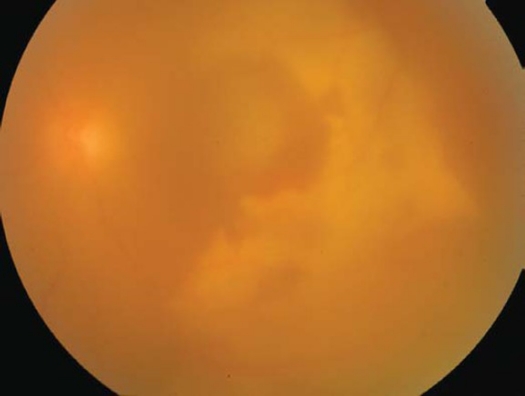
Color fundus photograph of the left eye showing vitreous hemorrhage, old and fresh subretinal blood.

**Figure 2:**
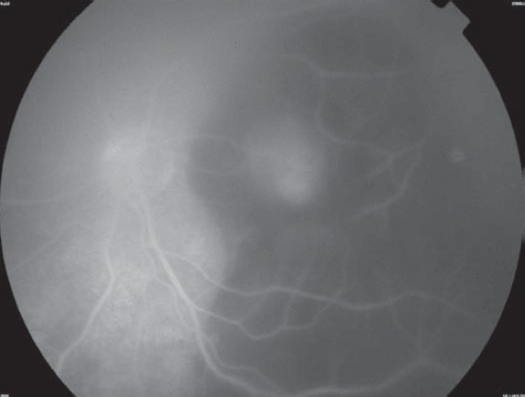
Intravenous Fluorescein of the left eye showing blockage of the dye by blood and dye leakage at late stage.

**Figure 3:**
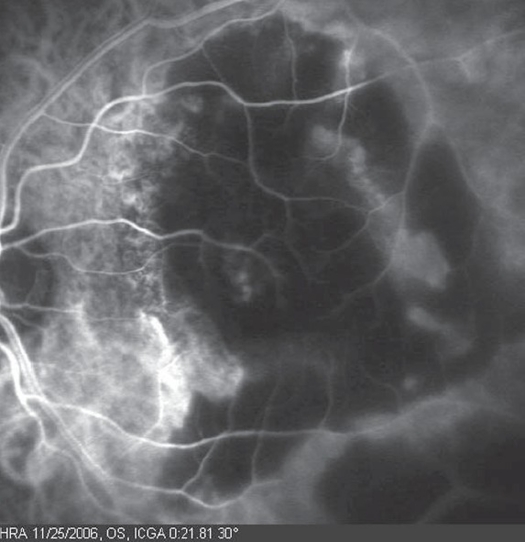
ICGA showing a branching of choroidal vascular network and collection of small polypoidal dilatation of the vessels.

**Figure 4:**
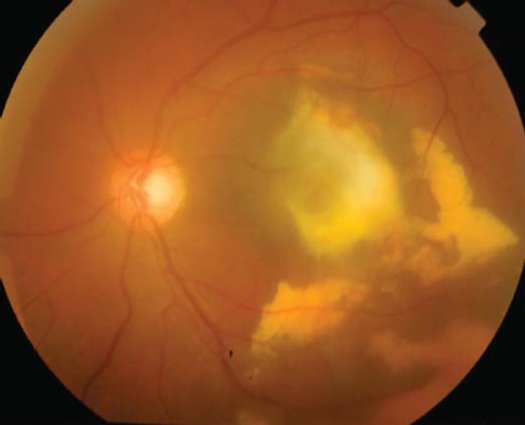
Color fundus photograph showing resolved hemorrhage and macular scar after 9 months of follow-up.

A 21-year-old healthy man suffered from sudden loss of vision in the left eye for aduration of 3 weeks. He denied any history of trauma. Best corrected visual acuity in the right eye was 20/20 and in the left eye was 1/200. Fundus examination of the right eye was normal; however, his left eye showed a significant vitreous hemorrhage obscuring the view with a massive sub retinal yellowish old blood in the posterior pole with epiretinal fresh blood on the fovea (Fig 1). Intra-venous Fluorescein angiography (IVFA) revealed fluorescence blockage by blood and dye leakage at a late frame that resembled the appearance of occult sub-retinal neovascularizations (Fig 2). Indocyanine green (ICG) test revealed a branching of choroidal vascular network and collection of small polypoidal dilation of the vessels (Fig 3). Based on these findings, the diagnosis of IPCV was made and the patient received photodynamic therapy (PDT after 3 weeks from date of presentatrion and then was repeated after 11 weeks from the first injection because the disease was still active. After a follow-up period of 9 months from first presentation the hemorrhage and sub macular fluid subsided leaving a macular fibroid scar (Fig 4). Final VA in the left eye was 20/200.

## Discussion

IPCV is a disease entity characterized by recurrent serous retinal leakage and hemorrhage in the elderly population and caused by vascular abnormalities in the inner choroids. The definition of IPCV has expanded over the past 10 years and the diagnosis is no longer restricted to specific demographic attributes or to specific retinal location. One large study done by Yannuzi et al^8^ showed that IPCV may occur in all races but with a predilection for heavily pigmented races. Women tend to be affected more often than men; the average age of onset of IPCV is considered to be 60 years. However, one case was reported in a 20 years old patient. IPCV is more commonly bilateral than unilateral and has prepapillary location. However, Sforzoliui et al^9^ found a preponderance of unilateral involvement and extra macular location of IPCV in Italian patients. Uyama and Kwork et al^10^,^11^ described the preponderance of men; unilateral involvement and macular location of PCV in Japanese and Chinese patients.

Indocyanine green test angiography is the best tool for diagnosis of IPCV. It usually shows a branching vascular network from the choroidal circulation and characteristic polypoidal, and aneurismal dilation at the terminals of branching vessels.^7^,^12^ The branching vascular network may last for a long period of time; however, the polypoidal dilations at the terminals of the network may change configuration; new dilations grow, while other regress.

Uyama et al^10^ described two patterns of polypoidal dilations in ICGA 1) a large solitary round aneurysmal dilation in which usually the patients with this pattern had a relatively favorable clinical course and 2) a collection of small aneurysmal dilations resembling a cluster of grapes. These lesions are usually active and tend to leak or bleed, and cause severe visual loss.

In agreement with Uyama, our case presents a group of small aneurysmal dilation from choroidal vessels resembling a cluster of grapes in ICGA, which were active and caused sever multilayered hemorrhages that resulted with poor vision and macular scarring.

One study^13^ described the clinical pathological correlation of IPCV in an enucleated eye with this diagnosis as dilated polypoidal lesions with large thin-walled cavernous vascular channels without a muscular layer that originated from branches of the short posterior ciliary's arteries.

The natural course of the disorder is not completely understood, but many patients have demonstrated chronic multiple recurrent serosenguineous detachment of RPE and neurosensory retina with long-term preservation of good vision. Some eyes do develop chronic atrophy and cystic degeneration of the fovea with severe visual loss. Other patients have experienced vitreous hemorrhage or secondary choroidal neovascular membrane with disciform scarring and profound loss of central vision.^8^ Uyama^10^ showed in his study that 50 percent of the patients with IPCV had a favorable course. The disease persisted and resulted in poor visual outcome in the remaining half of the patients.

A conservative approach, with observation, is adopted for asymptomatic IPCV unless the polyps result in exudative or hemorrhagic complication affecting central vision.

The use of PDT with verteporfine for the treatment of IPCV was described previously and showed a beneficial effect for the treatment of symptomatic IPCV.^14^–^19^

Quaranta et al^16^ and Rogers et al^17^ showed that PDT caused rapid visual improvement and occlusion of choroidal vascular abnormalities as was demonstrated by IVFA and ICG in retrospective analysis of 20 patients that received PDT for subfoveal IPCV. Spaid et al^14^ found that visual improvement achieved in 56.3 percent of their studied cases. However, in 31.3 percent of the cases vision remained the same and was decreased in 12.5 percent of the cases. Mean improvement in VA in these patients was noted to be2.4 lines. One patient had a transient increase in exudation and another developed branch retinal vein occlusion which did not affect the central vision. None of these patients developed any lasting complications from the treatment. Chan et al^15^ showed, in prospective study of 22 cases with symptomatic IPCV, stable or improved vision in 95 percent of the cases with mean visual improvement of 1.3 lines with complete absence of leakage on IVFA and total regression of polyps of ICG in 91 percent and 95 percent of the cases respectively after one year of follow-up. However, four eyes developed hemorrhagic complication after PDT which did not cause severe visual loss. Lea et al^18^ reported in his study that the visual stabilization or improvement was achieved in 78 percent of the patients and there was no recurrence detected with a follow-up period of 3 to 18 months after PDT. Mauget-faysse et al^19^ showed a significant improvement in the vision of all cases (30) with exudative IPCV after one year of follow-up. The serous detachment of the macula was completely resolved in 83.3 percent of the eyes and the polypoidal dilations were occluded in 73.3 percent of the cases.

In our case, the patient received PDT twice without significant improvement which was probably related to the severity of the disease at the time of presentation.

In conclusion, we described a complicated case of IPCV in a 21-year-old patient which is an unusual age of presentation for this disease and led to severe visual loss.
